# COVID‐19 effect on patients with noncommunicable diseases: A narrative review

**DOI:** 10.1002/hsr2.995

**Published:** 2022-12-15

**Authors:** Ahmad R. Al‐Qudimat, Mohamed B. Al Darwish, Mai Elaarag, Raed M. Al‐Zoubi, Mohamed Amine Rejeb, Laxmi K. Ojha, Abdulqadir J. Nashwan, Timoor Alshunag, Karam Adawi, Abdelfettah El Omri, Omar M. Aboumarzouk, Aksam Yassin, Abdulla A. Al‐Ansari

**Affiliations:** ^1^ Department of Surgery, Surgical Research Section Hamad Medical Corporation Doha Qatar; ^2^ Department of Public Health Qatar University Doha Qatar; ^3^ Department of Biomedical Sciences, QU‐Health, College of Health Sciences Qatar University Doha Qatar; ^4^ Department of Chemistry Jordan University of Science and Technology Irbid Jordan; ^5^ Nursing Department Hamad Medical Corporation Doha Qatar; ^6^ College of Medicine Qatar University Doha Qatar; ^7^ School of Medicine, Dentistry and Nursing The University of Glasgow Glasgow UK; ^8^ Center of Medicine and Health Sciences Dresden International University Dresden Germany; ^9^ Hamad General Hospital Hamad Medical Corporation Doha Qatar

**Keywords:** COVID‐19, effectiveness, epidemiology, mortality, noncommunicable disease, prevalence

## Abstract

**Background and Aims:**

On March 11, 2020, the WHO has declared COVID‐19 a global pandemic, affecting our day‐to‐day lives. Physical distancing and lockdown made significant obstacles to populations, particularly healthcare systems. Most healthcare workers were reallocated to COVID‐19 facilities. Noncommunicable disease patients were given low priority and are at a higher risk of severe COVID‐19 infection, which disrupted the treatment and disease management of these patients. This review aimed to assess the effect of COVID‐19 on different types of noncommunicable diseases and the severity it may cause to patients.

**Methods:**

We have conducted a review of the literature on COVID‐19 and noncommunicable diseases from December 2019 until January 2022. The search was done in PubMed and Cochrane for relevant articles using variety of searching terms. Data for study variables were extracted. At the end of the selection process, 46 papers were selected for inclusion in the literature review.

**Result:**

The result from this review found that the COVID‐19 pandemic has affected the efficiency of the patient's treatment indirectly by either delaying or canceling sessions, which solidified the need to rely more on telemedicine, virtual visits, and in‐home visits to improve patient education and minimize the risk of exposure to the patients. The major and most common types of noncommunicable diseases are known to be related to the severe outcomes of COVID‐19 infection. It is strongly recommended to prioritize these patients for vaccinations against COVID‐19 to provide them with the protection that will neutralize the risk imposed by their comorbidities.

**Conclusion:**

We recommend conducting more studies with larger population samples to further understand the role of noncommunicable diseases (NCDs) in this pandemic. However, this pandemic has also affected the efficiency of NCDs treatment indirectly by delaying or canceling sessions and others.

## 1 INTRODUCTION

On March 11th, 2020, the COVID‐19 global pandemic status was declared, having far‐reaching implications on our daily lives.^1^ Physical distancing and lockdown made significant obstacles to populations, particularly healthcare systems. Also, the lack of personal protective equipment, and more restrictions on healthcare providers and their contact with patients to reduce infection spread made it even harder on these patients. On the other hand, medical institutions were possible sources of infection which decreased the patients' willingness to seek care.^2^

Most healthcare workers were re‐allocated to COVID‐19 facilities. This caused the management of noncommunicable diseases (NCDs) to significantly scale down, especially during the initial pandemic outbreak, this caused NCD patients to be given low priority, and focused mostly on COVID‐19 patients. It has been reported that NCDs are increasing the risk of severe COVID‐19 infection conditions.^3^ Also, their treatment plans and management of their diseases may have been affected because of their limited access to hospitals or clinics due to the pandemic.

In normal situations, NCDs count for 7 out of 10 of the major causes of premature deaths.^4^, ^5^ In the 21st century, between the ages of 30 and 69 years, there has been around 15 million premature deaths caused from NCDs (38%, 15/40 million). In addition, 85% of premature deaths occur in middle and low‐income countries.^6^, ^7^ There are many different types of NCDs, however, the most common types are obesity, hypertension, diabetes, cardiovascular diseases (CVDs), chronic obstructive lung disease (COPD)/asthma, chronic kidney disease (CKD), and cancer. In fact, patients with obesity, diabetes, or CVD can have metaflammation and immunometabolism dysfunction, which can lead to disability, premature aging, and death.^8^ Lifestyle‐related diseases killed more people in the past than it is now. However, the disease burden of type 2 diabetes and obesity continues to increase. In the USA, obesity is believed to be the a major reason that life expectancy is decreasing.^9^

Most NCD medications play a role with the angiotensin‐converting enzyme 2 (ACE2) receptor. ACE2 is a protein receptor that permits the access of COVID‐19 virus into the cells and has been involved in the severity that diabetic and hypertensive patients experience when infected with the virus. The ACE2 is also found in the gastrointestinal tract, kidney, heart, and alveolar cells in the lungs. Angiotensin‐converting enzyme inhibitors (ACEis) and angiotensin receptor blockers (ARBs) are two types of renin‐angiotensin‐aldosterone system (RAAS) inhibitors found in medications.^10^ RAAS plays an important role in maintaining blood pressure. Angiotensin II is the effector molecule of RAAS, it is involved in the progression of CVDs, such as hypertension, myocardial infraction and heart failure.^11^ ACEis and ARBs reduce the activity of angiotensin II by elevating the expression of ACE2.^12^ Since ACEis and ARBs are found in the medications given to some NCD patients that have hypertension, diabetes, CVD, and CKD, it means that in these patients; the ACE2 activity is increased and may give COVID‐19 an easier access into the cell. Even though the effects of ACEis and ARBs in NCD patients with COVID‐19 are not conclusive yet, the results are promising and there is a great need to see their relationship to this virus.^10^

Herein, in this review, we will discuss how COVID‐19 infection plays a role in patients diagnosed with the different types of NCDs and the severity it may cause on them.

### 1.1 Literature search

#### 1.1.1 Information source and search criteria

A comprehensive search was performed in PubMed and Scopus databases between December 2019 and January 2022. The following keywords were used: (“covid 19” or “COVID 19” or “sars Cov 2” or “coronavirus” or “corona virus”) and (“non communicable diseases” or “non communicable disease” or “NCD” or “NCDs” or “chronic illnesses” or “chronic diseases”) as well as (“covid 19” or “COVID 19” or “sars Cov 2” or “coronavirus” or “corona virus”) and (“diabetes” or “cardiovascular diseases” or “hypertension” or “cancer” or “kidney disease”). Cochrane and Database of Abstracts of Reviews of Effectiveness databases were also checked for any systematic reviews. All non‐English publications were excluded. References from included papers were also screened for studies that might be relevant.

#### 1.1.2 Eligibility criteria

All articles assessing the management of COVID‐19 noncommunicable diseases were included. Comparative and noncomparative observational studies assessing epidemiological characteristics of comorbidities and associated with severe COVID‐19 disease with noncommunicable disease. Outcome measures were the following: 

1. Epidemiological characteristics
2. Associated with severe COVID‐19
3. Length of hospitalization
4. Intervention.

**Figure 1:**
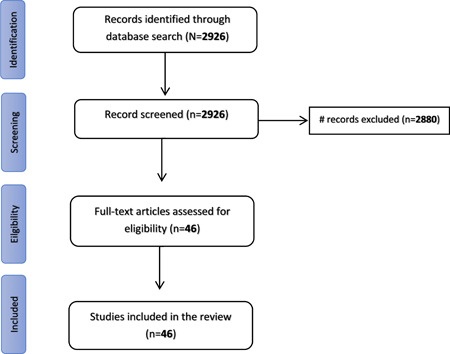
Flowchart of literature search

Figure 1, provides a flowchart of the selection criteria for the papers in this literature review. Initially, 2926 records were identified and after screening for titles, abstracts and full papers, 46 records were retained which were used in this literature review and which we elaborate on throughout this paper.

#### 1.1.3 Study selection and data collection

Two writers (A. R. Q. and R. M. Z.) screened and assessed all relevant article texts to ensure that they met the inclusion criteria. Where there were differences of opinion, the senior author (O. M. A.) was consulted until a consensus was established. We only included the most recent papers to minimize duplication of previously published studies by the same authors/institutions.

## 2 RESULTS

### 2.1 COVID‐19 and obesity

Eating habits have been changed during the pandemic, where people either lost or gained weight. This caused an obesity epidemic, which happens when a large number of the population gains weight due to the consumption of high caloric processed foods.^13^ This may also be related to stress, depression or other reasons caused by government restrictions.^14^ Simonnet et al. reported that infection with COVID‐19 was seen in people with a higher body mass index (BMI) causing to a greater probability of needing a ventilator.^15^ This means that more obese patients end up in the intensive care unit (ICU) compared to healthy weighted patients. Furthermore, a report showed the mortality risk was increased among younger COVID‐19 infected patients with a BMI ≥ 40 kg/m^2^.^16^

Higher mortality rates were also reported in 30 different studies that looked at obese patients infected with COVID‐19.^8^ Denson et al.^17^ reported that 88% of patients who complained of metabolic syndrome while infected with COVID‐19 were obese. Obesity may result in the diagnosis of diabetes, hypertension, CVD, stroke, or even cancer.^18^ In comparison to other patients, these patients have a higher risk of COVID‐19 complications and are more likely to require hospitalization and mortality. However, there is still a need for more research to better understand the mechanism between COVID‐19, an increased BMI, and obesity.^9^ (Table 1).

**Table 1:** Relevant research studies included in this review

Disease	No. of studies	Study design included
Obesity	8	Retrospective^4^
		Literature review^2^
		Cross‐sectional study^2^
Hypertension	15	Meta‐analysis^1^
		Review study^6^
		Retrospective study^4^
		Cross‐sectional study^3^
		Literature review^1^
Diabetes	19	Meta‐analysis^7^
		Review study^5^
		Retrospective study^4^
		Cross‐sectional study^1^
		Literature review^2^
Chronic kidney disease	6	Retrospective study^4^
		Literature review^1^
		Cross‐sectional study^1^
Cardiovascular disease	3	Meta‐analysis^1^
		RCT^1^
		Cross‐sectional study^1^
Chronic liver disease	5	Literature review^5^
Asthma	7	Literature review^7^
Cancer	5	Literature review^1^
		Cross‐sectional study^1^
		Retrospective study^3^

### 2.2 COVID‐19 and hypertension

Hypertension was a common comorbidity found in severe and fatal COVID‐19 cases.^3^ In literature, 97% of metabolic syndrome patients infected with COVID‐19 had hypertension, which may increase risk of hospitalizations and mortality.^17^

COVID‐19 virus enters the body and then the spike protein S attaches to the cells through the ACE2. The major prevalence in hypertension is that the RAAS in hypertensive patients is dysregulated and they usually take drugs that contain ACEis to regulate it, which may or may not increase the ACE2 activity.^6^, ^19^ Moreover, hypertension has been linked to immune dysfunction, which can lead to cytokine dysregulation. It may also cause systemic inflammatory response syndrome and acute respiratory distress syndrome (ARDS) that has been observed in severe COVID‐19 patients.^3^ A comparison was made between ARBs and ACEis with non‐RAAS inhibitors and it showed that patients using ARBs and ACEis both had numerically low mortality risks. (3.6% vs. 2.2%) (adjusted hazard ratio  0.85, 95% CI = 0.28–2.58 and *p*value = 0.774). This may be due to the sample size being small played by chance in both groups tested by Chao et al.^10^ There is still not enough evidence to conclude that ACEis and ARBs used by hypertensive patients can increase mortality and prognosis of COVID‐19. However, it has been recommended by the Council on Hypertension of the European Society of Cardiology (ESC) patients continue with their antihypertensive treatments and Gao et al.^10^ support this. When compared to patients who do not take any hypertensive treatments, results show that patients on hypertensive medications before admission had a lower mortality rate.^10^

Finally, it has been reported by Pranata et al.^20^ that hypertension has shown to have an association with bad risk outcomes (risk ratio [RR] = 2.1, 95% CI = 1.9–2.4), severity (CI = 1.7–2.5), ICU care (95% CI = 1.3–3.3, RR = 2.1), mortality (95% CI = 1.7–2.8, RR = 2.2), ARDS (95% CI = 1.1–2.4, RR = 1.6) and disease progression (95% CI = 1.5–6, RR = 3). Parveen et al.^21^ had similar findings: ICU care (95% CI 0.2–0.8, odd ratio [OR] 0.5), severity (95% CI 1.3–5.7, OR 2.7) and death (95% CI 0.3–0.7, OR 0.5).

### 2.3 COVID‐19 and diabetes

Several epidemiological reports consider diabetes as one of the key comorbidities linked to COVID‐19 and affecting its severity.^22^ Diabetes as a distinctive comorbidity is associated with increased infection of influenza, pneumonia, and acute respiratory distress syndrome, which leads to a three times higher (7.3%) fatality rate (2.3%) of COVID‐19.^3^, ^9^, ^23^, ^24^, ^25^, ^26^

According to several reports, the percentage of COVID‐19 patients who have diabetes ranges from 14% to 44%.^27^, ^28^, ^29^, ^30^ When epidemiologists compared diabetic and nondiabetic patients, they found that diabetic patients have a significantly reduced chance of survival or recovery, also they have a considerably higher chance of developing a serious disease progression.^2^ 92% of patients with metabolic syndrome had diabetes, these patients had higher rates of hospitalizations and deaths than others, whether they only had diabetes or a combination with other comorbidities.^17^

The ACE2 has a lower expression in diabetic patients because of glycosylation. As mentioned above, hypertensive patients have ACEis in their drugs which is also found in diabetic patients' drugs. Diabetic patients have very similar reactions to COVID‐19 as hypertensive patients because the drugs they use result in the same reactions in the body. Thus, using ACE2 stimulating drugs would give an easier entry for COVID‐19 into the cells and cause more severe and fatal diseases.^31^ It also shows that diabetic patients with both micro and macro vascular complications were related to an increased risk of insert of intubation and early passing on the seventh day of being hospitalized.^22^, ^24^

Several reports have shown the significant relationship between diabetes and their outcome, for example, Yan et al.^32^ reported that diabetic patients have a less survival rate than nondiabetic patients, also found RR of dying (3, 95% CI = 1.3–6.8). Similarly, based on another study in Mexico, diabetic patients infected with COVID‐19 have longer hospitalization and poor results, some existing research reviews support these conclusions.^33^ The risk of severity among diabetic patients infected with COVID‐19 is high and has been reported by Du et al.^34^ the results are: (RR = 2.1, 95% CI = 1.8–2.6), and risk of death (RD) (RR = 2.1, 95% CI = 2.6–3.8). In this context, Praveen et al.^21^ found that diabetes has a lower rate among the survivors (OR = 0.6, 95% CI = 0.4–0.9) with a strong association between diabetes and infection severity (OR = 1.7, 95% CI = 1.2–2.3). Another study conducted by Noor et al.^35^ reported a positive statistical association between COVID‐19 infected diabetic patients and mortality (RR = 1.9, 95% CI = 1.2–2.8).

Additionally, Lu et al.^36^ found that risk factors of mortality among diabetic patient and comorbidity are (OR = 3.7, 95% CI = 2.4–5.9). On another hand, Awortwe et al.^23^ reported the association with negative clinical outcomes of COVID‐19 patients and severity of infection (RD = 0.1, 95% CI = 0.01–0.09), admission to ICU (RD = 0.1, 95% CI 0.04–0.2), and mortality (RD = 0.1, 95% CI 0.1–0.2).

It is crucial that diabetic patients take better precautionary measures to reduce their risk of getting infected with COVID‐19, like social distancing, wearing masks, and hygiene. In addition, they should be taking their medications and regulating the glucose levels in their body which will then improve glycemia and boost their immune system response.^31^

### 2.4 COVID‐19 and chronic‐kidney‐disease

Recently, among all the NCDs, CKD has contributed to a rise in global mortality and morbidity. It is the fourth most common chronic illness that causes serious COVID‐19 infection outcomes.^33^ The COVID‐19 infection has been related to kidney dysfunction which then increases the risk of mortality.^37^ The challenges that CKD patients faced during the pandemic include operating healthcare services, available healthcare workers, and access to important medications and diagnostics, especially for those who require dialysis or kidney transplant.^37^ A study from India discussed the effect that the pandemic has done on dialysis patients, and it showed that approximately 30% of patients missed at least one session of dialysis and 3% required emergency dialysis.^38^

Any autoimmune disease, including kidney diseases, may be prescribed immunosuppression medications that work by lowering the immune system activity which makes it harder to fight off infections like COVID‐19.^39^ As mentioned previously, ACE2 is the binding site of COVID‐19 and in CKD patients, the kidney expresses ACE2 in the proximal tubules' brush border apical membrane that could help the virus enter and infect the glomerular endothelial cells.^40^

The effect of COVID‐19 on CKD has been rarely studied, however, it does seem to increase the severity of infection according to a retrospective study done in turkey by Otzurk et al.^41^ The study demonstrated the outcomes that CKD patients infected with COVID‐19 faced and it showed that CKD patients had significantly higher ICU admissions and mortality rates compared to the general population (114/289 [39.4%]; 95% [CI] 33.9–45.2; and 82/289 (28.4%); 95% [CI] 23.9–34.5). Similar results were shown in a meta‐analysis study from China (OR 3.03 [95% (CI) 1.09–8.47]).^40^ Overall, patients with CKD are required to take extra precautions to reduce their risk of getting infected with the virus and should be considered an important risk stratification for COVID‐19 models.^40^

### 2.5 COVID‐19 and CVD

Coronary heart disease, chronic heart failure and a history of cerebrovascular accidents were the most common types of cardio/cerebrovascular diseases found in severe COVID‐19 patients. However, the review by Gold et al.^3^ did not reveal an association between the two, this can be because CVD patients take specific medications that can alter the viruses' activity. A study by Ansa et al.^42^ showed that 57%–71% of CVD patients with angina pectoris, myocardial infarction, or stroke take aspirin regularly. Aspirin is an anticoagulant, but it also plays a role in hindering the replication of some types of coronaviruses. It shows that a soluble form of aspirin interferes with the viral protein and viral RNA synthesis to stop the replication transcription complexes of a virus.^17^ Another medication used by CVD patients is statin. Statin reduces the plasma concentration of a sensitive marker of systemic inflammation called C‐reactive protein.^43^ Therefore, statin can offer protection against acute respiratory distress syndrome when infected with COVID‐19.^3^

On the other hand, ACE2 is highly expressed in the cardiovascular system and more in patients with CVD. This high expression may cause infection or myocarditis while infected with COVID‐19. However, Gold et al.^3^ reported no statistical significance when comparing CVD or cerebrovascular disease in patients infected with COVID‐19, which might be due to the small sample size. More reports involving larger cohort are needed to draw further results.

### 2.6 COVID‐19 and liver disease

The liver remains one of the most vital organs in the human body, it plays an essential role in immunoglobulins production, and among other important functions, it supports the immune system.^44^ Liver disease causes approximately 2 million deaths per year globally between cirrhosis, hepatitis, and hepatocellular carcinoma.^45^ Since the emergence of the COVID‐19 pandemic, multiple studies reported the effects of coronavirus infection on patients with pre‐existing chronic liver disease (CLD). For instance, Marjot et al.^46^ conducted an international registry study and concluded that liver disease related to alcohol and baseline liver disease stage are found to be independent risk factors for death from COVID‐19. Patients diagnosed with CLD with cirrhosis have an elevated risk or mortality (32%) compared to patients without cirrhosis.^43^

A study found that 57.3% of COVID‐19 cases with pre‐existing liver disease were severe with a 17.7% death rate.^47^ Similar results can be seen in a systematic review by Khan et al.^48^ who concluded that liver disease patients are at an increased risk of dying from COVID‐19 (OR = 2.4, 95% [CI] 1.5–3.7). However, another systematic review found conflicting evidence, they did not find any significant association between mortality in COVID‐19 patients and CLD. Therefore, it is recommended conducting further studies to further investigate the association between CLD and COVID‐19.

### 2.7 COVID‐19 and asthma/COPD

The novel coronavirus targets the respiratory system, which causes acute respiratory disease that may lead to respiratory failure, pneumonia, and death.^49^ This raises major concerns about patients who live with CLD such as asthma and COPD. This should push researchers to study the interaction of these diseases with COVID‐19. One study undergone in Israel has conflicting evidence and found that asthma patients are not at a higher risk of hospital admission when compared to patients without asthma. In addition, a multivariate analysis study in Korea by Choi et al.^50^ also found no significant relationship between asthma and mortality or ICU admission in COVID‐19 patients (OR 1.317, 95% [CI] 0.708–2.451) and (OR 0.656, 95% [CI] 0.295–1.460). This study concluded that asthmatic patients are less susceptible to infection with the COVID‐19 virus which could be attributed to the lower expression of ACE2 found in lung tissue.^51^ As mentioned previously, ACE2 is the entry point of COVID‐19, when the activity of ACE2 is low, it lowers the probability of COVID‐19 entering the cell.

On the other hand, COPD is a disease that permanently affects lung function and it is a risk factor for developing pneumonia and the severe outcomes associated to it.^52^ According to a retrospective study in Spain by Graziani et al.,^53^ found that COVID‐19 patients with COPD have worse clinical outcomes when compared to people without COPD, such as mortality (3.4% vs. 9.3%: OR 2.93; 95% [CI] 2.27–3.79) with pneumonia being the number one cause for hospitalizations (59%) (31.1% vs. 39.8%: OR 1.57; 95% [CI] 1.14–1.18). Similar results can be seen in a systematic review by Alqahtani et al.^54^ with an increased risk of severe disease and mortality in COPD patients. Another retrospective study from Italy by Lacedonia et al.^55^ suggests that COPD patients are not at an increased risk of getting infected with the COVID‐19 virus. On the other hand, they had a higher all‐cause 30‐day death rate and poorer prognosis than their non‐COPD counterparts. However, after the multivariate analysis, it appeared to be related to the other comorbidities rather than COPD itself. Thus, it is recommended that COPD patients take extra protection measures against COVID‐19. Further studies to understand the effects of COVID‐19 on COPD and asthma patients with larger study groups are required to draw better conclusions.

### 2.8 COVID‐19 and cancer

Cancer has become one of the main factors contributing to mortality and morbidity in the last few decades, with various forms and types. Each type of cancer has unique morphology and pathogeneses. This possesses numerous challenges to both patients and healthcare systems.^56^ Those challenges only grew harder during the COVID‐19 pandemic, especially in an area where the outbreak causes major screening procedures and treatments to not be performed efficiently due to the social distancing protocols and to minimize the risk of exposure for the patients. This leads to delayed cancer diagnosis and treatment sessions and additionally makes enrolling in clinical trials more complex due to suspension or cancellation of trials.^56^ According to Lee et al.^19^, active cancer patients are 60% more likely to get infected with COVID‐19 compared to cancer free individuals, in addition to a 2.2‐fold increase in risk for those undergoing anticancer treatment. Cancer patients are not only more prone to catching the virus but are also associated with worse outcomes as stated in a retrospective case‐control analysis by Wang et al.^57^ In addition, a study from the United States found that patients with active cancers had higher rates of hospitalizations (47.46%) and deaths (14.93%) due to COVID‐19 when compared to the control group (12.39%) and (4.03%) respectively. This makes cancer patients as a high‐risk group that requires additional measures of protection against the novel virus.^26^ Another study made a comparison between hematological tumors and solid tumors, it came to the conclusion that hematological tumor patients have a higher mortality rate relative to patients with solid tumors when infected to COVID‐19 (33.33%).^58^ This confirms that cancer patients are one of the most exposed groups to suffer the devastating outcomes of COVID‐19 and highlights the importance of applying prophylactic measures to protect them from the disease.

## 3 DISCUSSION

People gain weight when the calories consumed is higher than the calories getting burned in the body, in other words, when the food exceeds the energy expenditure.^59^ Without physical activity and sitting for a long period of time contributes to the risk of weight gain.^60^ In the wake of the COVID‐19 pandemic, many countries have recommended that people stay at home as a primary means of limiting human exposure to the virus and thereby limiting its spread. As a result of decreased physical activity, changes in food consumption, and the stress involved with adjusting to a new situation, weight change may be a possibility during the quarantine period. According to a study conducted in Poland with 59,711 total subjects from 32 countries to examine the impact of COVID‐19's first lockdown on body weight, body weight changes were reported by 11.1%–72.4% of those polled, with the majority reporting an increase in body weight after or during the lockdown period.^61^ The most significant rise in body weight was discovered in an Iraqi study in which 45.6 percent of participants were between the ages of 21 and 30 years old.^62^ An Increase in the population's body weight during lockdown is due to a range of variables, including lifestyle, eating habits, and physical activity changes.^63^

In an astonishing nationwide broadcast, it is stated that, during the COVID‐19 outbreak, a bad prognosis was associated with the presence of a comorbid factor, one of which was very severe hypertension.^64^ According to the findings of the studies, anxiety and hypertension were highly related, and anxiety was found to be an independent risk factor for the development of incident hypertension.^65^ As per Zuin et al.^66^, a systematic evaluation of 419 individuals discovered that hypertension was the most common comorbidity associated with the virus. Patients with COVID‐19 infections with a high blood pressure had a significantly greater risk of death when compared to patients with normal blood pressure. Another study, conducted in January 2020, found that 11 out of 99 individuals with COVID‐19 died, with three of those patients having high blood pressure.^67^ Yang et al. support the aforementioned hypothesis, using data from 1576 COVID‐19 patients.^68^ When compared to patients who did not have severe illnesses, 2.36 is the OR of hypertension in severely sick patients. This indicates that hypertension may be a risk factor for patients who have severe illnesses. Yang et al.^69^ discovered that when infected with COVID‐19, hypertension increased D dimer, and as the ratio of neutrophil and lymphocyte increased, the mortality rate also increased.

In two big studies that included a total of 22,455 reported an increased mortality risk for diabetic patients infected with COVID‐19: (RR 2.12 [95% CI: 1.44–3.11], *p*value = 0.001; pooled OR: 1.90 (95% [CI] 1.37–2.64), *p* value = 0.001)

In another report that included 76 studies with a total of 31,067 patients, found results that were largely consistent with the above findings: diabetic patients infected with COVID‐19 had higher mortality rates (28.5% vs. 13.3%, respectively; *p* = 0.01) and a higher mortality risk (OR: 2.21 (95% CI: 1.83–2.66), both of which were statistically significant (*p* = 0.001) in comparison to those without diabetes.^70^, ^71^ It has been recommended by The Chinese Diabetes Society that COVID‐19 diabetic patients that are hospitalized receive personal blood glucose control and treatment strategies.^72^ Klonoff et al.^73^ stressed the significance of judicious use of glucocorticoids, increased blood glucose monitoring, increase interaction with health care professionals, and cautious discontinuation of ACEIs and ARBs. The seven treasure policy for diabetes control, according to Chinese's recommendations, should include blood glucose monitoring, health education, physical activity, healthy nutrition, standardized medicine, and mental health care.^74^ As of March 17, 2020, the median age of 2003 Italian patients who died after being infected with COVID‐19 was 80.5% (IQR 31–103), most of them being men (70%). There were 355 deceased diabetic patients who had their comorbidities recorded, with a prevalence rate of 35.5%.^75^

Infection with COVID‐19 can be mild or critical. It can cause a lot of damage to several different organs. No mention of COVID‐19's kidney involvement or the impact of pre‐existing kidney disease was made in early papers. Acute kidney damage and urine abnormalities were found in later research. Among Medicare beneficiaries, individuals receiving care for end stage kidney disease had a 3.5‐fold greater risk of death compared to those who did not receive care for the condition.^76^ According to researchers, other studies have found that patients on maintenance dialysis or kidney transplant recipients who develop COVID‐19 have a 25% case fatality rate.^77^, ^78^

In an international cohort of patients with CLD and COVID‐19 infection, Marjot et al.^46^ demonstrate that the severity of baseline liver disease is a significant predictor of prognosis in a significant proportion of cases. Specifically, they discovered that the severity of liver disease raised the risk of all major unfavorable outcomes, including hospitalization and death. Furthermore, it has been shown that CLD patients without cirrhosis live longer and die in the 8th decade of their life, however, mortality in CLD cirrhosis patients has shown to be distributed equally across different age groups, with a higher mortality rate in those under the age of 40. On the other hand, patients who had advanced liver disease after contracting the COVID‐19 infection had a particularly bleak outlook, with their chances of recovery diminishing as the illness progressed. In hospitalized patients admitted to the ICU with Child‐Pugh C cirrhosis, they had an overall survival rate of 46%, and the rate was even lower, 10%, in those who required invasive ventilation.^46^

COVID‐19 puts a major burden on COPD patients who are suffering from advanced disease severity. However, healthcare data show that the incidence of COVID‐19 in COPD patients has been relatively low, which goes “against all expectations” and “against all prognoses.”^79^ On the other hand, NCDs such as diabetes, heart disease, and hypertension have shown an increase in the COVID‐19 severity and frequency of infection. There was a low prevalence of COPD in COVID‐19 patients, however, high rates of severe complications (63%) and mortality (60%). This shows that COPD patients who get infected with COVID‐19 have a higher risk of complication and death.^54^ The majority of studies that reported COPD severity have characterized the cases based on ICU admission, significant oxygenation problems, required mechanical ventilation, or died as the result of the disease.^80^, ^81^, ^82^, ^83^, ^84^, ^85^, ^86^

Infection with COVID‐19 appears to raise the risk of mortality and severe sickness in cancer patients, independent of whether the malignancy is active, whether the patient is receiving anticancer treatment, or both.^87^ Zhang et al.^88^ published and presented their findings from a retrospective case study that included a total of 28 cancer patients who have been infected with COVID‐19. According to the results of the study, the rate of fatalities experienced by these patients was significantly elevated (28.6%), this is more than 10 times higher than the total numbers of COVID‐19 patients in China. Two papers from a single institution in New York city have talked about the outcomes of cancer patients. According to the findings of an aggregate‐level study conducted on 334 cancer patients treated at the Mount Silai Health system, the mortality rate was 11%, and the rate of intubation was also 11%.^89^ Despite the author's acknowledgment of a severity bias, the findings of a study conducted on 218 cancer patients treated at Montefiore Health revealed a case fatality rate of 28%. There was a death rate of 25% across the range for those who had solid tumors. COVID‐19 was related to a greater mortality (37%) in patients who had hematologic malignant tumors. Although breast cancer and genitourinary cancer had rates of 14% and 15%, they were associated with a considerably lower mortality rate when infected with COVID‐19 compared to other cancers.

## 4 CONCLUSION

After reviewing the available literature, the major and most common types of NCDs are directly related to the severe outcomes of COVID‐19 infection. However, the exact mechanisms that affect the course of disease remain a debate. Thus, we recommend conducting more prospective studies with larger population samples to further understand the role of NCDs in this pandemic

Many conclusions can be drawn from the results of our review. We can clearly see the close relationship between NCDs such as obesity, diabetes, hypertension, CKD, cancer, COPD, and asthma with COVID‐19 severe outcomes, where pre‐existing conditions make these patients highly susceptible to hospitalizations and even increase their risk of death according to the data presented in our review. This might be explained by the association of these diseases with ACE2 receptors that may be overexpressed in some types of NCDs, mostly in hypertensive and diabetic patients. This forms a pathway for the virus into the human body. However, research is still exploring this possibility and other possible mechanisms such as a cytokine storm. This pandemic has also affected the efficiency of NCDs treatment indirectly by delaying or canceling sessions, which solidified the need to rely more on telemedicine, virtual and in‐home visits to improve patient education and minimize the risk of exposure to the patients. Finally, we strongly recommend prioritizing people with NCDs for vaccinations against COVID‐19 to provide them with protection that can neutralize the risk imposed by their comorbidities.

### 4.1 Strengths and limitations

Our review provided research on the reasons behind the high susceptibility of NCD patients to COVID‐19. The researchers hypothesized two main things: (i) increased ACE2 (angiotensin‐converting enzyme 2) receptor expressions that facilitate the entry of the virus into the host body; and (ii) hyperinflammatory response, referred to as “cytokine storm.” In addition, this review points out that these transmission mechanisms are at play when it comes to all COVID‐19/NCD linkages. Finally, the literature review did not find any evidence that diabetes or hypertension‐related medications exacerbate the overall COVID‐19 condition in chronic illness patients. Based on this there are a few implications/policy recommendations that will stem from this research.

This review has a few restrictions that should be considered. First, the main included studies were retrospectives and cross‐sectional. Second, there were studies limited to adult humans than pediatric patients. Third, the main studies concerned DM and HTN more than other NDCs. Therefore, we need more comprehensive prospective studies to be conducted in this area to help with the better application of the results in the clinical treatment of these individuals and likely future pandemics of similar pathophysiology.

## AUTHOR CONTRIBUTIONS

**Ahmad R. Al‐Qudimat**: Conceptualization; data curation; formal analysis; methodology; writing – original draft. **Mohamed B. Al Darwish**: Data curation; methodology; writing – original draft. **Mai Elaarag**: Data curation; methodology; writing – original draft. **Raed M. Al‐Zoubi**: Conceptualization; methodology; supervision; writing – original draft; writing – review and editing. **Mohamed Amine Rejeb**: Methodology; writing – original draft. **Laxmi K. Ojha**: Data curation; methodology; writing – original draft. **Abdulqadir J. Nashwan**: Data curation; writing – original draft. **Timoor Alshunag**: Data curation; formal analysis; writing – original draft. **Karam Adawi**: Methodology; writing – original draft; writing – review and editing. **Abdelfettah El Omri**: Methodology; writing – original draft; writing – review and editing. **Omar M. Aboumarzouk**: Conceptualization; methodology; supervision; writing – review and editing. **Aksam Yassin**: Conceptualization; methodology; supervision; writing – review and editing. **Abdulla A. Al‐Ansari**: Methodology; supervision; writing – original draft; writing – review & editing.

## CONFLICT OF INTEREST

The authors declare no conflict of interest. Dr. Abdulqadir Nashwan is an Editorial Board member of Health Science Reports and a co‐author of this article.

## TRANSPARENCY STATEMENT

The corresponding author, Raed M. Al‐Zoubi, affirms that this manuscript is an honest, accurate, and transparent account of the study being reported; that no important aspects of the study have been omitted; and that any discrepancies from the study as planned (and, if relevant, registered) have been explained.

## Data Availability

The data that supports the findings in this study are available from the corresponding author upon reasonable request.
